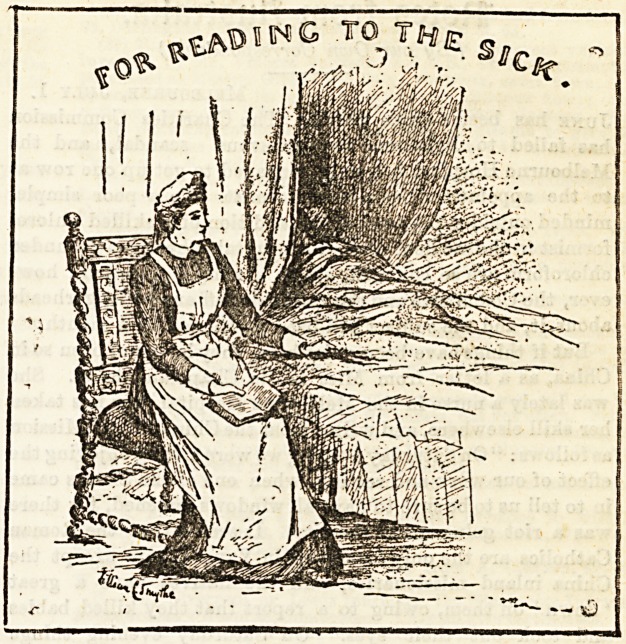# The Hospital Nursing Supplement

**Published:** 1891-09-12

**Authors:** 


### "The Hospital" Nursing Mirror.

Being the Extra Nursing Supplement of "The Hospital" Newspaper.

Contributions for this Supplement should be addressed to the Editor, The Hospital, 140, Strand, London, W.C., and should have the word "Nursing" plainly written in left-hand top corner of the envelope.

### En Passant.

THE PENSION FUND. — Last Friday evening an interesting meeting was held at the Trained Nurses' Club, 12, Buckingham Street, Strand, when Mr. George Pocock, Secretary to the Royal National Pension Fund for Nurses, gave an admirable address, in which he explained in a most clear and business-like manner the aims and objects of the Fund. A large number of nurses were present, and many of them gladly availed themselves of the opportunity of making enquiries about the pension fund with the intention of joining it.

GLASGOW QUEEN'S NURSES.—Last autumn the Glasgow Sick Poor and Private Nursing Association, 220, Sauchiehall Street, Glasgow, was visited by Her Royal Highness the Marchioness of Lorne, and inspected by Miss Paget, the Queen's Inspector for the Jubilee Nurses' Institution. It was found to be up to the standard required by Her Majesty for affiliation. Upon this, those nurses who wished the honour of being called Queen's Nurses and wearing the Victoria Badge sent in their names, only those engaged among the poor being eligible. The two superintendents and seven nurses have been accepted: Miss Wood (Lady Superintendent) and Miss White (District Superintendent), trained at Westminster Hospital; Nurse Allan, Edinburgh Royal; Nurse Sutherland, Glasgow Western; Nurse Towers, Paisley, N.B.; Nurse Neil, Royal Glasgow; Nurse Hunt, Royal Glasgow; Nurse Epps, Tottenham Infirmary.

MODERN MIDWIFERY.—Mrs. Withers, the wife of a labourer at Balderton, on August 14th sent for Mrs. Newstead to attend her during labour. Mrs. Newstead practices midwifery, has attended about twenty confinements, and charges a fee of 3s. 6d. The labour was a natural one, but the after-birth was fast, and "things began to go wrong." Mrs. Newstead sent for Mrs. Graves, a midwife, who said she had attended to over twenty cases, and charged a fee of 5s. Things continued to go wrong, though Mrs. Graves states that she did her best, and always had had luck before. Then Mrs. Graves sent for Mrs. Cullen, also a midwife, who had "done for a good many," and "always had very good luck," and "charged 5s." Still things continued to go wrong; in fact, the life of the poor mother was ebbing slowly away, and when the hour of death was at hand, a doctor was sent for. He arrived when all was over. In his evidence before the coroner he stated, "I have not the slightest doubt that if I had been sent for earlier the woman would have been now alive." It seems useless to comment on these cases; they are frequent and painful, but the public turns a deaf ear. The subject of cruelty to children is dilated on in drawing-rooms, and ladies weep and give golden guineas, but for some reason the lives of poor mothers in their hour of peril is not a fashionable or sentimental subject. The Committee of the Midwives' Registration Bill find it impossible to rouse interest or obtain funds. Yet surely to rid the country of these horrible Sairey Gamps, who double and make more bitter, the curse of Eve is work which should arouse the energy of any woman who has any sympathy for her sex. The full particulars of this case can be found in the *Newark Herald* for August 22nd, and the picture of those three "midwives" round the wretched mother is one which for horror and pathos is almost unequalled.

SHORT ITEMS.—When the nurses of the Leeds Borough Hospital are nursing typhus cases the Matron sees that they get a daily drive.—Princess Louise will attend the October meeting of the Scottish Branch of the Queen Victoria Jubilee Institute.—The nurses and probationers of Salford Hospital have sent a formal complaint of the food supplied to them to the Committee.—*Hearth and Home* had an article on "Nursing," on August 27th, and an article on "Women Chemists," on September 3rd, both under the heading of "What to do with our Daughters."—Our New Zealand querists will have an additional interest now in emigrating —the women of New Zealand have now the suffrage and are eligible to sit in Parliament.

STOCKTON NURSING ASSOCIATION.—On August 26th Lady Londonderry made an interesting speech at Stockton in aid of the District Nursing Association, which she established. Lady Londonderry said: "I have been kept fully informed of every detail in connection with the scheme, and have marked with extreme satisfaction as well the growth of it as the interest which has been evinced in it by all classes in the town. I rejoice in the fact that among the Council are included five members who are representatives of the working classes, for when we consider that this Association was formed mainly for the benefit of those classes, it is satisfactory to think that they can thus render us material assistance both by their advice and by practical knowledge of the wants of those whom it is our desire to benefit. I think I should not be exceeding my duty in moving the adoption of the report if, in addition, I were to mention that the Tramcar Company have agreed to give free passes for the nurses. I must express on our behalf generally our grateful thanks to those who have generously assisted us both in money and kind, and I have no doubt that if the same zeal and generosity are displayed in the future as have been in the past we shall have the satisfaction of seeing our Association steadily increasing in utility, and, by its means, in alleviating many cases of distress and proving itself of inestimable benefit to the town of Stockton."

THE NURSE AND THE UNDERTAKER.—We have received from one of our nurse-readers a letter sent her by an undertaker, who says: "I have been recommended some very good funerals by medical, monthly, and other nurses, the profits of which we divided. Would it suit you to recommend me on the above terms? My equipments are of the very latest and most approved designs, and my charges will be found to compare favourably with those of any other firm. Distance no object." What a subject this offers for the pen of James Payn, who delights in grim jokes at the expense of the noble profession of nursing! To us, however, the serious side of the question presents itself, for it is not the first time we have come across an unholy alliance between nurse and undertaker, and have been humiliated to think that women practising a healing art could thus dare to profit by death. Of course were it known that a nurse worked in conjunction with an undertaker her career would be absolutely ruined. No good nurse would ever dream of such a thing, and we now warn Matrons and heads of private nursing institutions to keep a close watch over the less reputable members of their staff in case the love of gain prove too much for their sense of decency and honour. Also if other cases of this sort come to our notice we shall publish the names of the firms which dare to make these proposals

### Lectures on Surgical Ward Work and Nursing.

By Alexander Miles, M.D. (Edin.), F.R.C.S.E.

### Lecture XXXV.—KNIVES.

There are very many forms of knives used in surgery, and I cannot attempt to do more than describe a few of the more common and generally useful of these. (*a*) *Scalpels* are of various sizes and shapes, have a short, broad blade, with a sharp point, and are chiefly used in surgical operations, in dissecting out tumours, &c., where very great care is necessary, to avoid injuring important strictures, such as blood vessels and nerves. (*b*) *Bistouries* are somewhat like scalpels, but on the whole are longer, especially in the blade, which is narrow in proportion to its length. Altogether a bistoury is a more "graceful" instrument, if one may use the term. They are used in making long incisions in the skin, or in performing small amputations, such as fingers, toes, foot of a child, and so on. *Varieties* are indicated by their names, *e.g.*, straight sharp-pointed bistoury; straight probe-pointed bistoury; curved sharp-pointed bistoury; curved probe-pointed bistoury. Those with probe points are used in parts where there is danger in introducing a sharp-pointed instrument, for example, in splitting up a long sinus near any large bloodvessel or other important structure. (*c*) *Amputating Knives* are of very various sizes and patterns, some having a single cutting edge, others cutting with both edges; some with sharp points, some rounded, and so on, varying according to the operation and the taste of the operator. *Syme's Amputating Knife* was used by that surgeon in performing his amputation at the ankle joint. It is a short, strong, broad-bladed knife, with a very thick back and a large handle, like all other amputating knives. (*d*) *Abscess Knives.* (1) Syme's abscess knife has a short, somewhat sickle-shaped blade, which is bevelled off at the sides. When thrust into an abscess, the point always tends to make its way to the surface again. (2) *Paget's Abscess Knife* consists of a thin, narrow, straight blade, attached to its handle by a thin metal stalk. (3) *Von Graefe's Cataract Knife* is used by some in opening small abscesses. It is particularly suitable for children and nervous women, as its size does not alarm them. (*e*) *Tenotomy Knives*, as their name implies, are used for cutting tendons, the operation being done subcutaneously. They may be sharp or probe pointed, are short, thin, and narrow. They only cut with one edge. Of course, the probe-pointed one can only be used after the skin wound has been made by the other. It is used as a protection against wounding blood-vessels, &c. (*f*) *Hernia Knives* are not often used by most surgeons. Their function is to divide the constructing band in cases of strangulated hernia, and for this purpose they are fitted with a long, curved, rounded stalk, about four inches long, having a very short cutting edge situated about an inch from the point, which is also blunt. On the back of the stalk there is a rough area, by which the surgeon may determine exactly the position of the cutting portion of the edge. The illustrations are used by kind permission of Messrs. J. Weiss and Sons and Messrs. Maw, Son, and Thompson.

### The Princess of Wales and the Nurses.

Photographs are pouring in for the screen which the First and Second Thousand nurses have decided to offer for the acceptance of the Princess of Wales on her next birthday. As not more than 1,000 photographs can be placed on the screen those nurses who wish to show their appreciation of the Princess's kindness to them should send carte and postal order for 2s. immediately, to Screen, care of Miss Pritchard, The Lodge, Porchester Square, London, W., or they may be too late. Postal orders from the following Nurses are acknowledged: E. Gosling, A. M. Belton, H. Newman, E. Franks, L. F. K. Bromfield, L. M. E. Cripps, C. A. Corbell, E. Winter. M. A. Wylie, M. Backworth, C. M. Martin, E. L. Maur, M. Ridsdale, E. Barnes, E. Custance, H. Kew, Grist, M. A. Harper, A. Harrison, E. M. A. Sherring, E. North, J. Porley, F. Shaw, F. Stockwell, S. Bellamy, C. Hutchinson, M. Calver, E. Gates, E. Whitehead, M. Edmonds, P. Kirk, Baskin, J. Solby E. Wills, Batchelor, A. Woods, M. Arnold, H. Winter, S. Edwards, M. Scott, L. Barton, S. Baxter, B. Chapman, L. J. Groves, Cowdell, A. Griffith, S. Watson, A. Latter, A. Newport, A. Thorpe, E. A. Mackaness, M. A. Bliroom, H. E. Elliott, M. McCartney, F. L. Saunders, H. Cole, C. Barton, E. Langley, E. Bellamy, M. E. Macdonnell, L. Robbins, E. A. Smirby, L. A. Bacon, S. Walker, J. Gilston, L. Nelson, E. M. Burgess, E. Jeffrey, E. Mills, M. Williams, H. Larsen, E. Parker, A. Phillips, C. Charlton, C. Roulston, M. Turner, L. Wing, L. Tidey, J. Landsborough, Proviss, S. Piddman, A. Snook, E. Bacon, L. Mawn, C. Lisle, S. Davis, B. George, Wakeling, Hutton, J. Histed, Gioney, H. Walker, E. Bagg, E. Barrow, P. Brady, M. Lee, Goldfinch, A. Chaffe, E. Dowse, F. Sadler.

### The Island of Gigha.

An advertisement appearing in these pages for a district nurse for the Island of Gigha recalls a memory of a pleasant holiday spent on that spot—a spot so little known that a few particulars here may interest others than those replying to the advertisement. Gigha is not so inaccessible as St. Kilda, of which Miss C. G. Furley lately gave an account; it is only two hours' sail from Tarbert, lying between Cantyre and Islay. There is no pier, but a small boat goes out daily to meet the steamer, if the weather permits. Gigha is seven miles long, and is a fertile and prosperous little island; there are four hundred inhabitants, and, as the nearest doctor is at Tarbert, and cannot be got under about thirty hours' notice, the nurse will have plenty to do, and it is essential she should be thoroughly competent in midwifery and pleasant and resourceful in all her ways. Water in Gigha is carried from the nearest spring, sanitary arrangements there are none, and many of the houses have only mud floors and windows which will not open. All this matters not in the summer, when the sea winds blow softly and the sunshine blazes on the brilliant heather; but in winter and in sickness there is scope for all nursing art and all womanly tact and sympathy. Perhaps only a Scotchwoman used to Highland ways could competently deal with the difficulties which will meet the district nurse of Gigha; but an English hospital nurse might well envy the lot of one spending her life in that pure atmosphere, and able constantly to see the glory of the sun-set behind the purple hills of Jura. "Island of God" is one of the meanings given of the word "Gigha," and truly to those who know its rocky headlands and sandy bays, who know its kindly inhabitants and all the legends which linger about its shores, the name is not inappropriate. May Gigha secure a devoted and clever nurse, able to appreciate the privilege which gives her noble work in a beautiful land.

### Appointments.

[It is requested that successful candidates will send a copy of their applications and testimonials, with date of election, to The Editor, The Lodge, Porchester Square, W.]

Adelaide Hospital.—Miss Rose Maud Bansk, trained at the London Hospital, and late Receiving Room Sister, has been appointed Lady Superintendent of the Adelaide Hospital; and Miss Pauline Chapple, trained at the London, and Staff Nurse there, has been appointed Night Superintendent at Adelaide. They sail this week, and the good wishes of many go with them.

Hong Kong.—Nurse Bertha Taylor, of the London Hospital, has been appointed Nurse at the Government Hospital, Hong Kong.

Monsall.—Miss Plowman, who was trained at St. Bartholomew's Hospital, and is at present Sister of a surgical ward of twenty-eight beds, at Pendlebury Children's Hospital, has been appointed Superintendent of Nurses at Monsall Fever Hospital, in succession to Miss Calvert.

Prince Alfred Hospital, Sydney.—Miss McGahey, late Matron of the Carrington Centennial Hospital, has been appointed Matron of the Prince Alfred Hospital—one of the most important and best managed institutions in Australia. Miss McGahey trained at the London Hospital and acted as Sister Cotton there.

Strafford Infirmary.—Miss M. E. Parker has been appointed Night Sister to the Infirmary, Strafford. She was three and a-half years at Monsall Fever Hospital, with charge of wards, and has just finished her two years' general training at the Royal Infirmary, Newcastle-on-Tyne.

Taunton Hospital.—Miss M. B. Wilson, trained at the London and late Sister Queen, has been appointed Matron of this hospital.

### PLAIN SAILING.

It is pleasant on a fine day to watch a vessel sail gently down a tidal river to the ocean. The sun shines, the wind is fair, she goes with the stream, nothing hinders or opposes her. The man at the helm keeps a lazy watch, just enough to prevent her grounding or running foul of any other craft; there are no signs of danger, all is peaceful and serene. Apparently no care is taken of the vessel, but is it really so? A wise captain will have everything ready for a emergency even when they seem most secure, for many a good ship has foundered because no preparation has been made for a possible danger.

There are times in our lives when we, too, find it all plain sailing; health, comfort, success in life seem to be our happy lot. A sudden illness assails us, or we lose our friends and our children are taken from us; then everything will depend on what we have taken aboard during our voyage, whether or not we shall reach the post we are sailing for. I hope that post is Heaven, and that we have been practising patience, and perseverance, and obedience, and all good virtues in order to get there. Because, if we have been keeping a watch over ourselves in health, we shall be the better able to bear our troubles and afflictions in sickness. For instance, if we have been obedient, we shall not grumble at the rules and restrictions so necessary in illness; if we are impatient when well, we shall not bear our bodily pains quietly, but groan and toss and tumble, and think ourselves very illused. If this has been our habit till now, it will be hard to break ourselves of it when our hearts are sore and our bodies racked with pain. Still we will not despair, but strive to get with us the Pilot who can guide us through the trials and sorrows of this world, for with Christ on board we shall soon find ourselves at the haven where we would be. It is far better to be guided by Him than to have our own way, to drop on aimlessly through life. A few storms, a few tears, these will strengthen us for future struggles in the voyage of life. Remember the beautiful story of the fishermen on the Sea of Galilee. They were crossing the late at night carrying our Lord with them, who was sleeping peacefully, when suddenly one of the fierce storms so frequent in that country came down upon the ship and filled it with water so that they were in jeopardy. In their terror they woke Him and called on Him to save them from perishing; and with one command—"Peace, be still"—there was a great calm. Christ can still the tempest and storms of our lives now as easily as when He was on earth. Let us not forget that He is always with us, and though He appears to sleep, yet we have One Who can make the winds of passion in our hearts to cease and the waves of trouble to be still.

### Notes from Australia.

*(By our Own Correspondent.)*

MELBOURNE, JULY 1.

JUNE has been a quiet month. The Charities Commission has failed to light on any grievous scandal, and the Melbourne Hospital has only managed to get up one row as to the appointment of a chloroformist. To a poor simple-minded outsider the need of the addition of a skilled chloroformist to the medical staff of a hospital where deaths under chloroform are so common, seems a simple question; however, the Committee and the medical staff are at loggerheads about it, and the subject had been postponed for a month.

But if things have been quiet here, they have not been so in China, as a letter from Miss Lizzie Chapman shows. She was lately a nurse in the Melbourne Hospital, but has taken her skill elsewhere, and writes from the China Inland Mission as follows: "On Saturday evening we were sitting enjoying the effect of our work and resting, when one of the Sisters came in to tell us to be sure to have all windows fastened, for there was a riot going on in the city. It seems that the Roman Catholics are the only foreigners in Yang-Tchow except the China inland missionaries, and the natives have a great 'down' on them, owing to a report that they killed babies and took out their eyes. On Saturday evening things reached a climax. The mob of Chinamen went up and attacked the place, breaking windows, smashing in doors, and doing all the damage they very well could. I heard this morning that they set fire to the building, and some of it was destroyed. We sat in our comfortable room trying to read, but not managing very well, for we could hear the shouts, shrieks, and yells of the mob. Gradually we became aware that the voices were coming nearer, and presently the whole mob gathered in front of our house. Ours is a front window, and got a full share of sticks and stones; but the shutters are strong, and stood it very well. Just try and imagine our position. Twenty-four girls, only three or four of whom could speak the language, and the rest having been in China only a month or less. No Englishman within miles and miles, for there are no other foreigners in the city. Shut up in a rickety old house with a mob of raging Chinamen outside, ready to do anything to get us out of the city." However, all ended well, and these brave women are working away, trying to heal both bodies and souls of the "heathen Chinee."

The Women's Hospital has been honoured by visits from Madame Sarah Bernhardt and Lady Hallé. The former had the temerity to state that the purity of the air in the wards and the general sanitary condition of the institution were superior to that of any hospital she had ever visited in France. Miss Ballantyne, the head nurse, has resigned. The Committee of the Convalescent Home for Women have set apart two beds at the home for hospital nurses requiring a rest.

The death of Dr. Beaney makes a vacancy on the Melbourne Hospital staff, for which there will be many competitors. The deceased gentleman amassed considerable wealth during his thirty-three years of life in the colony. It is understood that the bulk of his estate will be divided among the charitable institutions of Melbourne and Canterbury, England, the deceased gentleman's native town. His diplomas and all the presentation addresses and other marks of esteem which have been given to him are left by him to the mayor of Canterbury. Some of these were received for services rendered in the Crimean War.

Dr. Stuart Stephenson, M.B.C.M. Edinburgh, has been appointed resident Medical Officer to the Homœopathic Hospital.

Miss Farquharson, Matron of the Alfred Hospital, at the late Committee meeting submitted a report giving details of the recent examination of pupil nurses, which she deemed highly satisfactory. The conduct and general behaviour of the pupils had been good.

Mr. W. G. Brett, Inspector of Charities, furnished the following report:—

I have the honour to inform you that on the 23rd ult. I completed the inspection of the Alfred Hospital, on which date there were 125 patients in the ordinary wards, namely 62 males, 57 females, and six children; in the paying patients' wards four males and four females, and the total number of typhoid cases 16. The accommodation is for 72 males and 72 females, and for infectious cases 48 males and 24 females, or a total of 216. The list of the staff furnished to me showed a total of 75 resident and non-resident employés, including 28 class 'B' pupils in training, 15 of whom are in receipt of wages. The books and accounts for the year ended 30th June, 1890, were duly audited and certified to as correct. The cost per head is £75 5s. 6d., £7 10s. 7d. more than the mean cost per head for all hospitals, which is £67 14s. 11d. It is worthy of commendation that the endowment funds have been used in the extension of the hospital to meet the public requirements, and render the building suitable for the purposes for which it is intended. The nurse training school has been unable to do more than meet the demands of the hospital of late years and precluded the hiring-out of any nurses. I have pleasure in certifying to the good order, cleanliness, and efficiency of this fine institution, and in view of the increased expenditure when the number of inmates will be greater and the cost of administration higher in proportion, there is a good case for generous public support and favourable consideration of the claims of the hospital.

The following is from the *South Australian*: "The Board of Management of the Adelaide Hospital have reluctantly accepted the resignations of the Superintendent of Nurses (Miss Thwackthwaite) and the Night Nursing Superintendent (Miss Burke). These ladies arrived at the institution some time ago from England, having been specially sent out, and not intending to remain permanently, their purpose being to obtain fresh experience. With that object they will after leaving Adelaide go on to Melbourne and Sydney, eventually returning to London. They will stay in Adelaide three months longer, whilst arrangements are being made for the appointment of their successors. The Board expressed in the highest terms their appreciation of the capacity of the ladies for organisation and of the excellent management which has characterised their term of office. It has been decided that as the work to be done is of a special character, requiring considerable experience, two successors shall be obtained from England. Miss Lückes, one of the most distinguished ladies in her particular line, and holding a responsible position in the London Hospital, has been asked to recommend two suitable ladies for the vacancies, and in this matter she will act in concert with the Agent-General." London Hospital Nurses now hold all the good appointments in Sydney and Adelaide; let us hope they will soon get a foothold in Melbourne. Miss McGahey, the first Matron of the Carrington Centennial, who had all the furnishing and starting of that big institution has done her work well, but now goes elsewhere. Miss Noble at the Children's Hospital continues her good work. Dr. Constance Stone and Dr. Stella M. Taylor are two Australian women of whom we are proud, and to whom we look more since we lost Dr. Mary A. M. Knight.

### Presentation.

PHILLIPS' MEMORIAL HOSPITAL, BROMLEY, KENT.—Miss Evelyn Davey (trained at the London Hospital 1884 to 1886) who has resigned the Matronship here after 18 months' service, was, on her approaching marriage, presented by the Committee with a complete set of table cutlery and electro-plate.

### Wants and Workers.

*Sister Garrad* asks for any old perambulator with wheels and springs good for the use of invalid children. Address, Convalescent Home, Lambourne, Romford.

*Nurse H.* acknowledges with thanks 1s. from Nurses Lambert and Williams, 1s. from A. S. A., and 2s. from Alnwick.

### Everybody's Opinion.

*[Correspondence on all subjects is invited, but we cannot in any way be responsible for the opinions expressed by our correspondents. No communications can be entertained if the name and address of the correspondent is not given, or unless one side of the paper only be written on.]*

### WATER BEDS.

"NURSE L. H." writes: I have read with great interest your notes and queries on water beds, and should like to report my experience in contrast to "Sister's" remarks in your last issue. The water bed I now have in use came into my care in April, 1889. My then patient used it for eight days, when I emptied it and brought it with me to my present case, refilled it with pure pump water (warm) on the 14th of the same month, and once added a pail-full of warm water. The bed was emptied for removal on May 4, 1891, when "Sister" may be surprised to hear that the water was as free from smell as when put into the bed over two years before. If a disinfectant must be used, which I consider unnecessary in an air-tight article, let it be Condy's Fluid, which is inodorous, not carbolic acid. Of course I often wash the outside of the bed with disinfectants and regulate the temperature with hot-water bottles.

### THREE MONTHS' CERTIFICATES.

"S." writes: After reading "Revelations about Registrations" in THE HOSPITAL, I should like to express my opinion regarding a certificate I got at a lying-in hospital in October, 1884. In my mind the value of it is not worth the parchment it is written upon. It was signed by two medical men, who knew nothing whatever about me. I never spoke to either of them, in fact, the one doctor I never even saw, as he did not attend the hospital during the period I was there. I can only reflect upon the so-called training and certificate with contempt. My impression of the whole arrangement was that, when they had got your fee, £10 for eight weeks, their interest was very much at an end. According to the system there were certainly too many pupils admitted to act fairly towards them. I began my nursing career June, 1875, thus had had over nine years varied experience as a private nurse. It was only for that special branch of the profession I desired to gain knowledge. I was quite shocked at the ignorance and deficiencies of some whom I met there, and who were taken on by institutions and sent out as thoroughly efficient nurses.

### POOR SPINSTERS.

"A RICH SPINSTER" writes: I notice in your article of August 8th, on "The Royal Red Cross," a statement that the Nursing Sisters are "only poor spinsters." Allow me to inform you that they are among the richest of spinsters, and, furthermore, that they do not grudge their decoration to the married lady, who, after the one great display in becoming such, has such very occasional opportunities of showing her pluck. I grant you she outshines the Nursing Sister there. The usefully and congenially employed spinster and the happy married woman are about equal; of the unhappy married ones, how many would think themselves poor could they become spinsters again, and of the poor spinster (aimless and unoccupied), how many are happier for getting married?

### Notes and Queries.

### Queries.

(42) What are the proper strengths of perchloride of mercury and carbolic acid, as used in the London lying-in hospitals?—*Obstetric Nurse.*

(43) Will any doctor tell me if filters for water are advisable; if so, which is the most recommended, and where it can be procured?—*C. E. J.*

(44) What is the last resource allowed to a nurse in a case of post-mortem hæmorrhage, after the expulsion of the placenta?

(45) Wanted the names of institutions in or near London which would receive children with mental ailments, particularly a boy, aged eleven, blind, and mentally deficient.

(46) Can any nurse give me the address of cheap comfortable apartments at Bournemouth, for a nurse?—*Nurse E.*

### Answers.

(37) The Alpha Syringe, continuous flow, can be had from Mr. Gibson, 93, Darlington Street, Wolverhampton. Price 7s. 6d.

(37) Higginson's Syringe. used with a glass recurrent vaginal tube, invented by Mr. Vincent Jackson, F.R.C.S., of Wolverhampton, and purchased at Messrs. Gibson and Co's., chemists, same town, is one of splendid value, for its great advantage (continuous flow); otherwise nothing surpasses "The Donehe." Mr. Vincent Jackson, Waterloo Road, Wolverhampton, would, I am sure, be pleased to give you full instructions as to the usage of his invention. It is not costly.—*A. Pountney, L.M., Dublin.*

(38) A. L. had better write to Miss Lobb, Convalescent Home, Lambourne, Romford. She takes care of such cases at a moderate charge.

*Private Nurse.*—You are eligible to join the Pension Fund. Write to the Secretary, Royal National Pension Fund for Nurses, 8, King Street, Cheapside, London, E.C., and ask him to send you a pamphlet and particulars.

*C. Mc,K.*—We think nurses have been asked for quite enough lately; let the scheme rest for the present.

*N. J. M.*—Presentations are not formal matters, nor dependent on the religious question at all. We have always found that a gentle manner and an endearing presence and good work, were rewarded in Catholics and Protestants alike.

*Whitestone.*—If you watch the advertisements, you will see London hospitals occasionally advertising for nurses, but not often. Still, none of them have a hard and fast rule on this subject. Free training in midwifery can be had from the Workhouse Infirmary Nursing Association; again we must refer you to our advertisements.

*Miss Hale.*—Your proposal is good, but cannot be taken into consideration just now; we have so much on hand.

*J. E. Edeson.*—Send a stamped addressed envelope to Miss Hicks, Nurses' Co-operation, 8, New Cavendish Street, London, W. We know of no Co-operation in Liverpool.

*Scarboro'.*—The ear-caps can be had from A. Claxton, 62, Strand, W.C. Price 3s. 6d.

*B.*—The Liverpool or Leeds Infirmary, the Winchester Infirmary, or Addenbrooke's Hospital, Cambridge. They all give certificates.

*Fenheall.*—Nursing in India is connected either with the Countess of Dufferin's Fund, or Lady Roberts' Fund. You will find particulars of the last in THE HOSPITAL for February 7th, 1891. The Secretary of Lady Dufferin's Fund is Colonel John Robertson, 17, Inverness Terrace, Hyde Park. Next week we hope to give an article on nursing in India.

*A. L.*—There is no published list of Continental hospitals. Accounts of many of them appeared in *The Sunday at Home* in 1888. Perhaps Mrs. Brewer, 21, George Street, Hanover Square, W., would give you information if you asked her a definite query.

*E.C.*—You can compete for three prizes if you like. We hope you will, and take them all.

(40) Apply to the Fir's Home, Bournemouth, St. Mary's Home, High Street, Hastings (Homœopathic, 12s. a week), St. Luke's Lodge, Higher Lincoln Road, Torquay (12s. a week), or, most likely of all, St. Raphael's Hospital, Worthing (16s. a week).

*To Correspondents.*—Many letters are unavoidably held over this week.

*Mrs. Benwell.*—Sister Frances has left England; her address is St. Luke's Home, Vancouver, British Columbia.

*Nurse Corner.*—Socks safely received.

### Amusements and Relaxation.

### SPECIAL NOTICE TO CORRESPONDENTS.

**Third Quarterly Word Competition, commenced July 4th, 1891, ends September 26th, 1891.**

Competitors can enter for *all* quarterly competitions, but no competitor can take more than *one first prize* or *two* prizes of any kind during the year.

Proper names, abbreviations, foreign words, words of less than four letters, and repetitions are barred; plurals, and past and present participles of verbs, are allowed. Nuttall's *Standard* dictionary only to be used.

The word for dissection for this, the ELEVENTH week of the quarter, being

"MADRAS."

| Names. | Sept. 3rd. | Totals. | Names. | Sept. 3rd. | Totals. |
|---|---|---|---|---|---|
| Paignton | 44 | 297 | Christie | — | 44 |
| Psyche | 46 | 300 | Dulcamara | 42 | 287 |
| Hope | — | 47 | Nurse J. S. | 39 | 292 |
| Lightowlers | 48 | 314 | Qu'appelle | — | — |
| Wizard | — | 179 | E. M. S. | — | 68 |
| Wyameris | — | 46 | Jenny Wren | 47 | 283 |
| Dove | — | 46 | Carpe-diem | — | 65 |
| Punch | — | 181 | Grannie | — | 36 |
| Ivanhoe | — | — | Nurse G. P. | 28 | 172 |
| Tinie | — | 93 | Goodnight | — | 122 |
| Agamemnon | 46 | 305 | Gamp | — | 100 |
| Nurse Ellen | — | 86 | Charity | — | 104 |

### Notice to Correspondents.

Agamemnon.—No dissection of "Woodcock" received.

All letters referring to this page which do not arrive at 140, Strand, London, W.C., by the *first post on Thursdays*, and are not addressed PRIZE EDITOR, will in future be disqualified and disregarded.

N.B.—Each paper must be signed by the author with his or her real name and address. A *nom de plume* may be added if the writer does not desire to be referred to by us by his real name. In the case of all prize-winners, however, the real name and address will be published.

### Thrown Away.

"Let knowledge grow from more to more."

—*Tennyson.*

"Nell," said Dolly Strangways, "this is our last day in Oxford; let us make the most of it, for to-morrow we shall be again in the wards on duty, our very thoughts, not to speak of our time, no longer our own property. Shall we go on the river in the canoe?"

The sisters had never trusted themselves in the Canadian canoe, since the evening when their nerves had been shattered by their adventure with the rat that took refuge with them, and was received so inhospitably.

"Well?" Dolly went on. Nell wavered, hesitated, and, of course, was won.

"You might as well invite a man to go with you!" said a voice from behind a newspaper; "let's make a water-party, and I'll get Bob to come, too."

"Stupid thing!" was Dolly's cousinly rejoiner; "how can four go in a canoe, Harry?"

"Tell you what, though," said Harry, "two can walk on the bank, and two can paddle; we could take it turn about, do you see?"

"That would be nice!" put in Nell, with decision; so the thing was settled, and by-and-by the merry foursome set out in the sunshine, jesting and laughing, for they were all great chums.

"Now," said Dolly, who had elected herself commander-in-chief of the expedition, "I'll take Nell across to the opposite bank, and then, Harry, I'll come back for you; and you two can stroll along or race along, which you please, while Bob and I paddle our canoe. Come along, Nell; get on board. No rats in the hold, this time, my dear!"

Across the sunlit river sped the white-clad, laughing girls, and when Dolly presently returned for Harry, she found him lustily singing "Twickenham Ferry" to greet her.

"I say, Dolly," he paused in his song to remark, after she had shipped him, "the river's uncommonly deep here."

"Yes, it is," calmly rejoined Dolly; "fifteen feet at least, I believe; it's owing to the rapidity of the current."

"Well, just be careful, my dear girl, for perhaps you don't know that Bob can't swim."

"Then he has no right to come on the water," promptly returned Dolly, "especially in a cockle-shell of this sort."

"That's his affair," observed Harry. "You can't very well tell a man he'd better keep to the land until he knows how to conduct himself in the water as well as on the water. Here we are! Steady now!" and in a few minutes more Harry was safe on the bank, waving his straw hat to the departing ferrywoman as she paddled back again.

Walking up and down, smoking the cigar he had just lighted, Bob awaited the returning canoe. Bob was a man, the chief feature of whose character was self-possession, or, as we islanders love to call it, British *aplomb.* Calm and well-balanced, his mind was not given to lose its equilibrium on any provocation. But, like Achilles of old, we each have a weak point, a vulnerable spot, and Bob was so heedless as to be spending a great portion of his visit to Oxford on the river without possessing the necessary safeguard—the power of swimming.

"Come along, sir," cried Dolly, as she neared the shore. "Quick! Those two are off, pegging away for their lives, to race us, I believe!"

"Oh, they are, are they?" said Bob, cheerfully; "well, we'll see who'll win," and he stepped off the side into the middle of the canoe, before Dolly had time to hold on to the bank to make safe. Instantly, after the manner of such crafts, the canoe turned right over, and the two, Bob and Dolly, were capsized into the middle of the stream. There was a swish; a sudden darkness; a feeling as if the river were running down their throats; and then Dolly, an expert swimmer, came to herself. I must help Bob, was her first thought; Bob can't swim; and she quickly made for the bank, from which she hoped in some way to save her companion. Bob, on his part, retained his senses so far as to strike out as he best could, in his ignorance of how it should be done, for the shore. It had all happened in a moment, as accidents usually do. The tremendous splashing of the water fell on the ears of Nell and Harry, who rushed back in horror.

"Lay hold of the boat!" shouted and shrieked Harry and the two girls. Bob, though half afraid the canoe might possibly turn over again and suck him under, obeyed orders. His dread was realized; in a few seconds more the unmanageable craft turned another somersault, righting itself and riding blithely on the glinting ripples of the tide in its proper position.

But where was Bob?

A deadly, icy shiver seized the three on the bank as their straining eyes failed to see him rise again to the surface.

"He's drowning! Oh, save him! Help!" screamed Nell.

"Good Heavens!" muttered Harry, and his face grew chalky-white.

But Dolly said never a word; only flinging off her hat, she went to throw herself again into the river to swim to the rescue.

"Are you crazed?" shouted a stern voice, and Harry's strong arm forced her back, holding her in spite of her frenzied struggles. "Do you want to throw away your life, too? He's sucked under and drawn away by the current, God help him! What can either you or I do?"

Then Dolly threw herself on the grass, face downwards, moaning in anguish for the bright young life gone out before her eyes.

"Can *nothing* be done?" cried Nell, wringing her hands frantically. "How can you stand there, Harry, and not raise a hand?"

"What on earth *can* I do?" Harry wiped the great drops from his brow, for his utter helplessness was no light thing for a man to bear. "Shall I plunge in after him?" he hoarsely asked. "I cannot get at him, for he's gone down like a stone, or is a mile away by this time. But I can throw my life into the river; perhaps, I'd better!" and he began to tear off his coat.

"No, No!" Nell seized his sleeve. "But let us call somebody; let us do something!" But there was nothing to be done, though helpers began to hurry up in numbers as the news spread.

When the canoe was pulled up on the bank, someone looking it over pointed out the cigar that Bob had taken from his lips, in the water, and never losing his presence of mind, had carefully wedged it in the seat underneath the inverted boat, to which he tried to cling for salvation, but in vain.

Late in the evening, after the sun went down, the body, with its life thrown away, so to say, was got, entangled under some willows miles off, the victim of a foolhardiness that daily tempts men and women to play with an element over which they have not learned the control.

"Oh, if I had but stayed by him, in the river, and tried to hold him up?" sobbed Dolly as her tears fell on the motionless form, deaf and indifferent to her voice in its icy death-sleep, as it had never been in life.

Then, tender hands drew the girl away, and wise words told her that no puny efforts of hers could have saved the strong man.

## Figures and Tables

**Figure f1:**
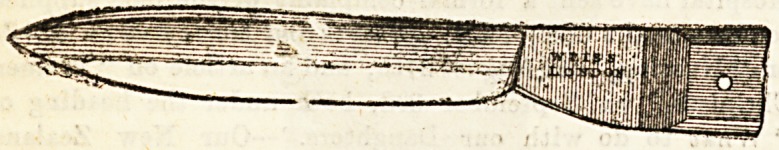


**Figure f2:**
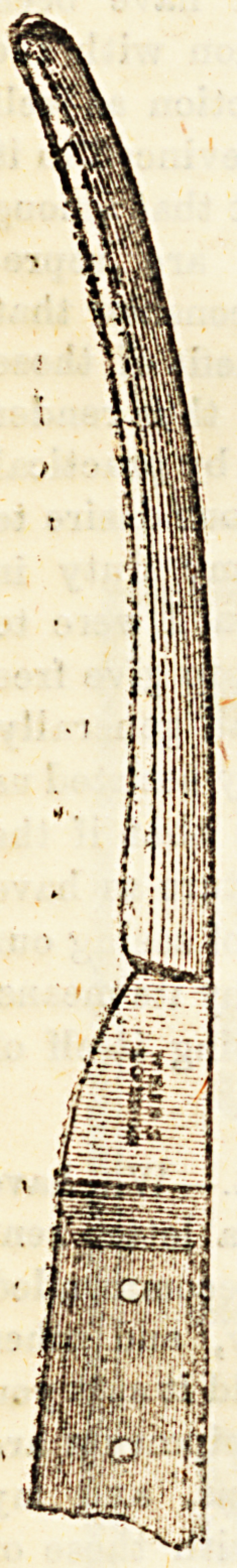


**Figure f3:**
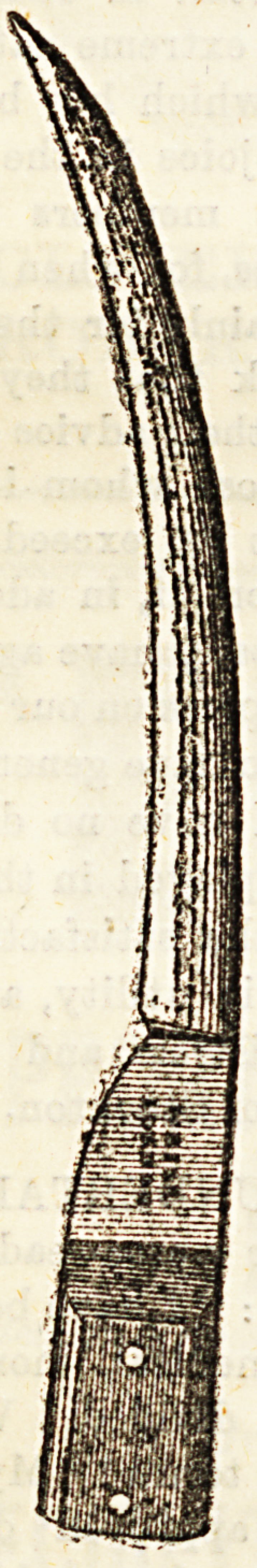


**Figure f4:**
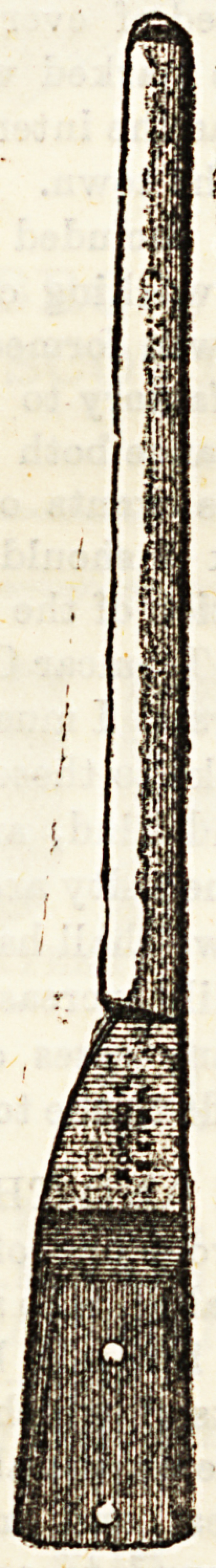


**Figure f5:**
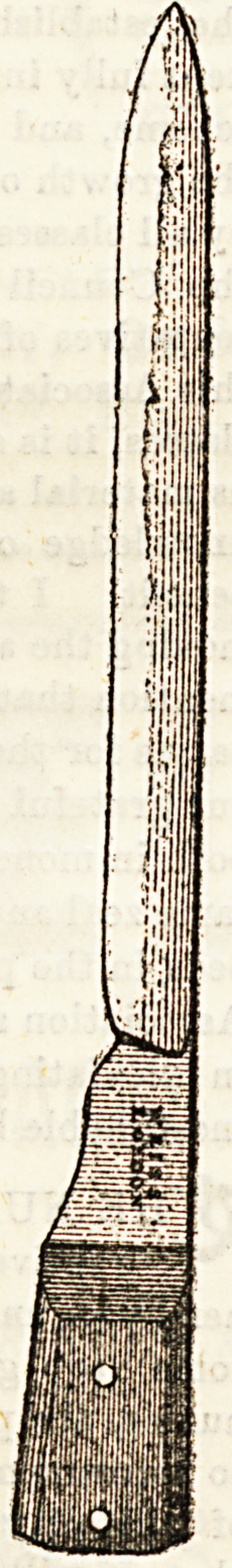


**Figure f6:**
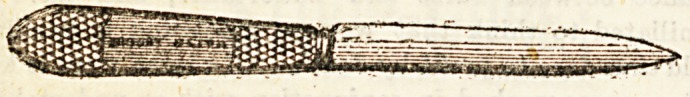


**Figure f7:**
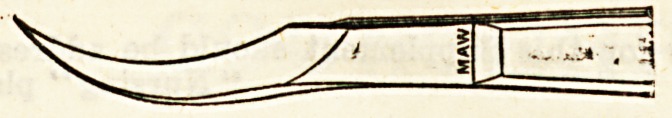


**Figure f8:**
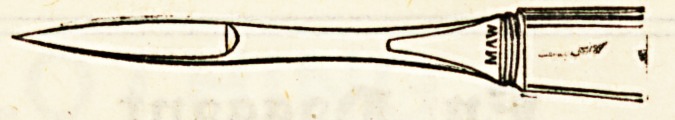


**Figure f9:**
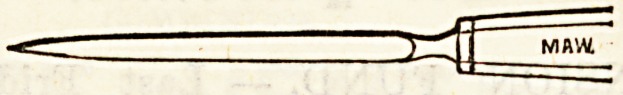


**Figure f10:**
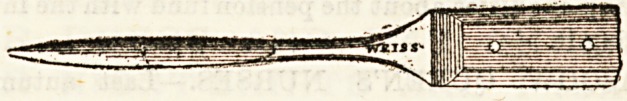


**Figure f11:**
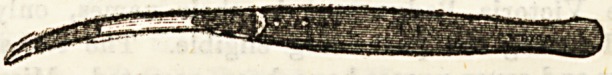


**Figure f12:**